# A Rare Case of Neuropathic Pain in Cutis Verticis Gyrata: A Review of Contemporary Literature

**DOI:** 10.7759/cureus.69939

**Published:** 2024-09-22

**Authors:** Vaibhav Oberoi, Kevin Morris, Inderjit Singh, Apindervir Kaur Mann, Gurpreet Kaur

**Affiliations:** 1 Department of Neurology, Government Medical College and Hospital, Amritsar, Amritsar, IND; 2 Department of Physical Medicine and Rehabilitation, University of Missouri School of Medicine, Columbia, USA; 3 Research, Society for Brain Mapping and Therapeutics, Los Angeles, USA; 4 Department of Research and Development, Aklun Biotech LLC, Nagpur, IND; 5 Department of Research and Development, Morris Lifesciences, Innovation and Research Center, Nagpur, IND; 6 Department of Internal Medicine, Government Medical College and Hospital, Amritsar, Amritsar, IND

**Keywords:** cutis verticis gyrata (cvg), dermatology, multimodal pain management, neurocutaneous disorder, neurological complication, neuropathic pain, scalp deformity

## Abstract

Cutis verticis gyrata (CVG) is a rare benign neurocutaneous condition marked by thickened scalp folds resembling cerebral gyri and sulci. It has been classified into primary essential, primary non-essential, and secondary types. The primary essential type is idiopathic, and the primary non-essential type may be associated with neurological or ophthalmological complications. Secondary CVG occurs more commonly than primary CVG, affects both genders at any age, and can be associated with endocrinal disorders, tumors, inflammatory diseases, or genetic conditions. For every 38.46 male cases, only one female case is known. This report aims to inform about a rare case of primary CVG in a 35-year-old female patient presenting with neuropathic pain localized to the affected area, a complication not previously documented in the literature. Heightened sensitivity to light touch and pinprick sensation in the high parietal scalp region was indicative of neuropathic pain. This report also highlights the importance of a multidisciplinary approach to treatment, including pharmacological treatment modalities such as analgesics and non-pharmacological management such as desensitization techniques and physical therapy. Critically, it is necessary to regularly follow up and perform proper clinical evaluation and screening to improve the quality of life for patients with CVG.

## Introduction

Cutis verticis gyrata (CVG) is a benign but rare condition identifiable by thickened folds and grooves of the scalp mimicking the surface of the brain. It is also known by other names such as paquidermia verticis gyrata or bulldog scalp syndrome. CVG can be classified into three types based on the lack of or the presence of any underlying cause [1]. It can present independently but is often seen in patients with other underlying ailments. It has been classified into three types: primary essential, primary non-essential, and secondary. The primary essential is idiopathic affecting only the scalp, while the primary non-essential can be associated with neurological disorders such as mental retardation, epilepsy, or ophthalmological manifestations [2].

For every 38.46 male cases, only one female case is known [3]. Primary CVG generally occurs in post-pubertal men aged less than 30 years, with an increased occurrence seen in patients suffering from mental disorders [4]. There is a lack of knowledge in the understanding of the pathogenesis of primary CVG. Researchers have postulated a hormonal influence as one of the causes since this condition is usually seen in post-pubertal men [1,5]. Secondary CVG, while affecting both males and females, is known to occur more commonly than primary CVG [6]. Secondary CVG is generally considered a representation of the underlying cause, such as due to a tumor, genetic condition, endocrinal disorders, or inflammatory diseases, and needs to be correlated to the associated pathophysiology of that specific underlying cause.

This case report aims to shed light on a novel case of association of CVG and neuropathic pain in a female patient in her mid-30s who was diagnosed with CVG and was experiencing neuropathic pain in the affected scalp region. After a thorough review of the literature, we found no other known documented case having similar symptoms of neuropathic pain and CVG association.

## Case presentation

A 35-year-old female presented to the outpatient unit of the Department of Neurology at our hospital with significant discomfort and pain in the parietal area for the past five to six weeks, described as burning and tingling sensations exacerbated by touch and movement. The patient mentioned that she noticed scalp deformity since childhood. On examination, the patient exhibited pronounced cutaneous hypertrophy and furrows over the vertex of the scalp, characterized by excessive folding and thickening of the scalp skin resembling the convolutions of the cerebral cortex consistent with a diagnosis of CVG. There were no signs of inflammation or infection.

The patient reported having unpleasant sensations such as burning and tingling and noticing discoloration of skin of the scalp area. Further neurological examination revealed heightened sensitivity to light touch and pinprick sensation with a ball of cotton wool in the affected scalp region indicative of allodynia, all suggestive of neuropathic pain. Other neurological examinations were insignificant. Imaging studies such as non-contrast computed tomography (NCCT) of the head were performed to check for and possibly rule out any underlying intracranial abnormalities contributing to the scalp deformity and to assess any structural changes in the scalp and underlying tissues. NCCT of the head showed evidence of diffuse thickening of the scalp region in bilateral high parietal area regions (left more than right) with marked ridges and furrows seen within it. The maximum width of ridges was 17mm, and the maximum depth of furrows was 15mm. This thickening was noted to be involving the dermis and subcutaneous region. Multiple specks of calcification were seen within this thickening. The brain parenchyma was normal in signal intensity with normal gray-white matter differentiation. An X-ray of the cervical spine was performed, which showed cervical straightening with paraspinal muscle spasm. Upon detailed past medical history, the patient reported having a history of drainage of purulent fluid once from the scalp folds, which was subsequently treated by another physician at the local village clinic with systemic antibiotics. We requested for any lab reports or histopathological reports from that visit, which the patient was unable to provide.

Laboratory tests, including complete blood count, differential leukocyte count, random blood sugar, hemoglobin A1c (glycated hemoglobin, glycosylated hemoglobin, HbA1c, or A1c), liver function tests, renal function tests, thyroid function test, lipid profile, chest X-ray, and electrocardiogram were within normal limits. Other general physical examinations were also insignificant.

**Figure 1:**
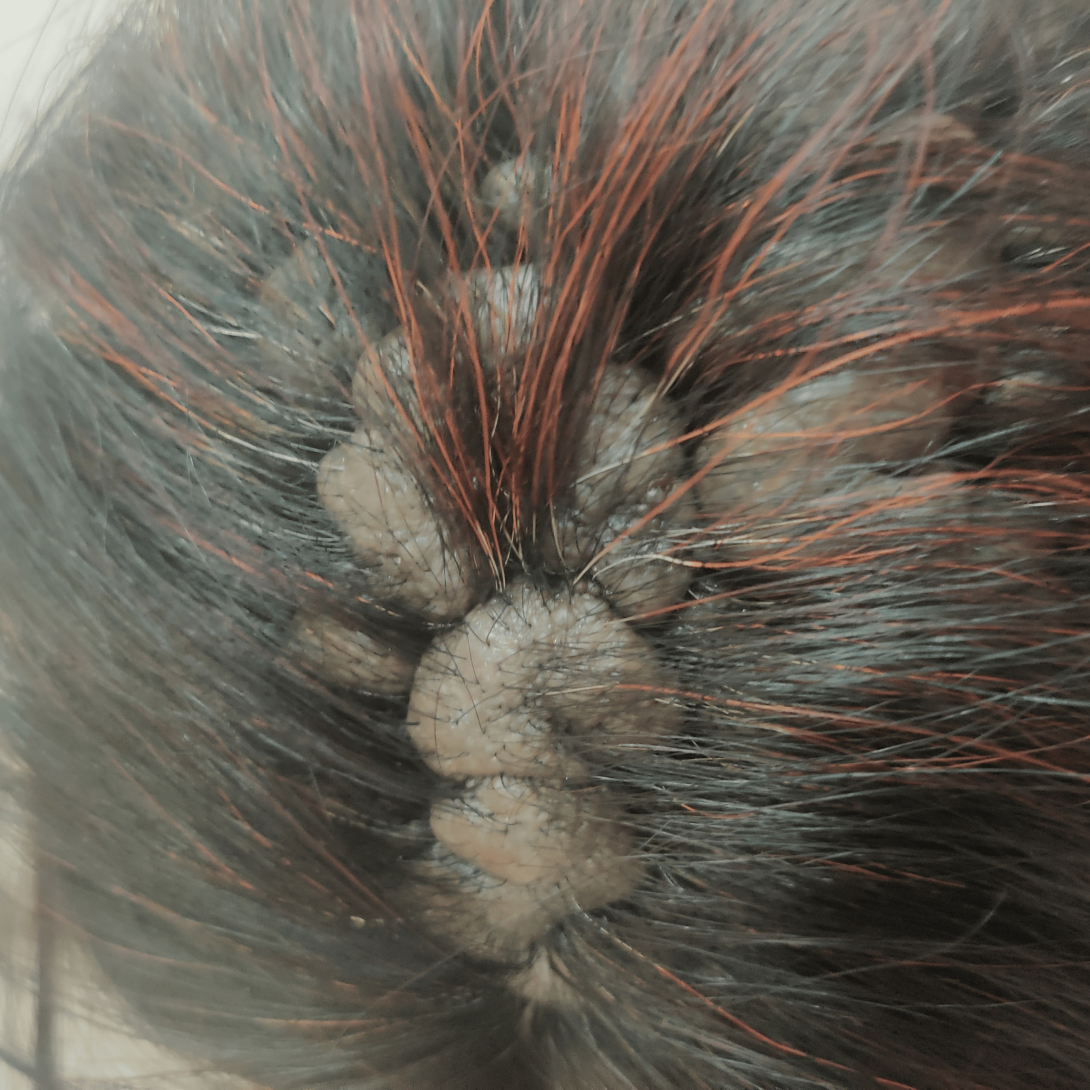
Scalp of a 35-year-old female patient with intense scalp neuropathic pain and deep, symmetrical cerebriform folds in the parietal areas, typical of cutis verticis gyrata. Image showing gross deformity in the high temporoparietal area. The photographic image taken by Dr. Vaibhav Oberoi after verbal consent was obtained from the patient during the visit to the outpatient clinic. All authors confirm the originality of the image.

The management focused on symptomatic relief of the neuropathic pain using pregabalin 75 mg (Pregalin-75 capsule) once before sleeping at night, along with non-opioid analgesics and application of topical lidocaine as needed. Non-pharmacological approaches, such as desensitization techniques (including gentle head massage, and ice pack application), and physical therapy (yoga postures and counselling) were also employed to alleviate pain and improve the quality of life for the patient. Surgical options for cosmetic correction were discussed but deferred due to the preference of the patient and the risks associated with the procedure. The patient continues to be monitored regularly in the dermatology and neurology outpatient clinics. There has been substantial improvement in tolerance to pain intensity and daily functioning, as reported by the patient with the current management regimen. Long-term follow-up is planned to assess the stability of symptoms and to consider further interventions as needed. Figure 1 is a photograph of the scalp of the patient showing gross deformity in the high temporoparietal area, as typically seen in CVG.

## Discussion

**Figure 2:**
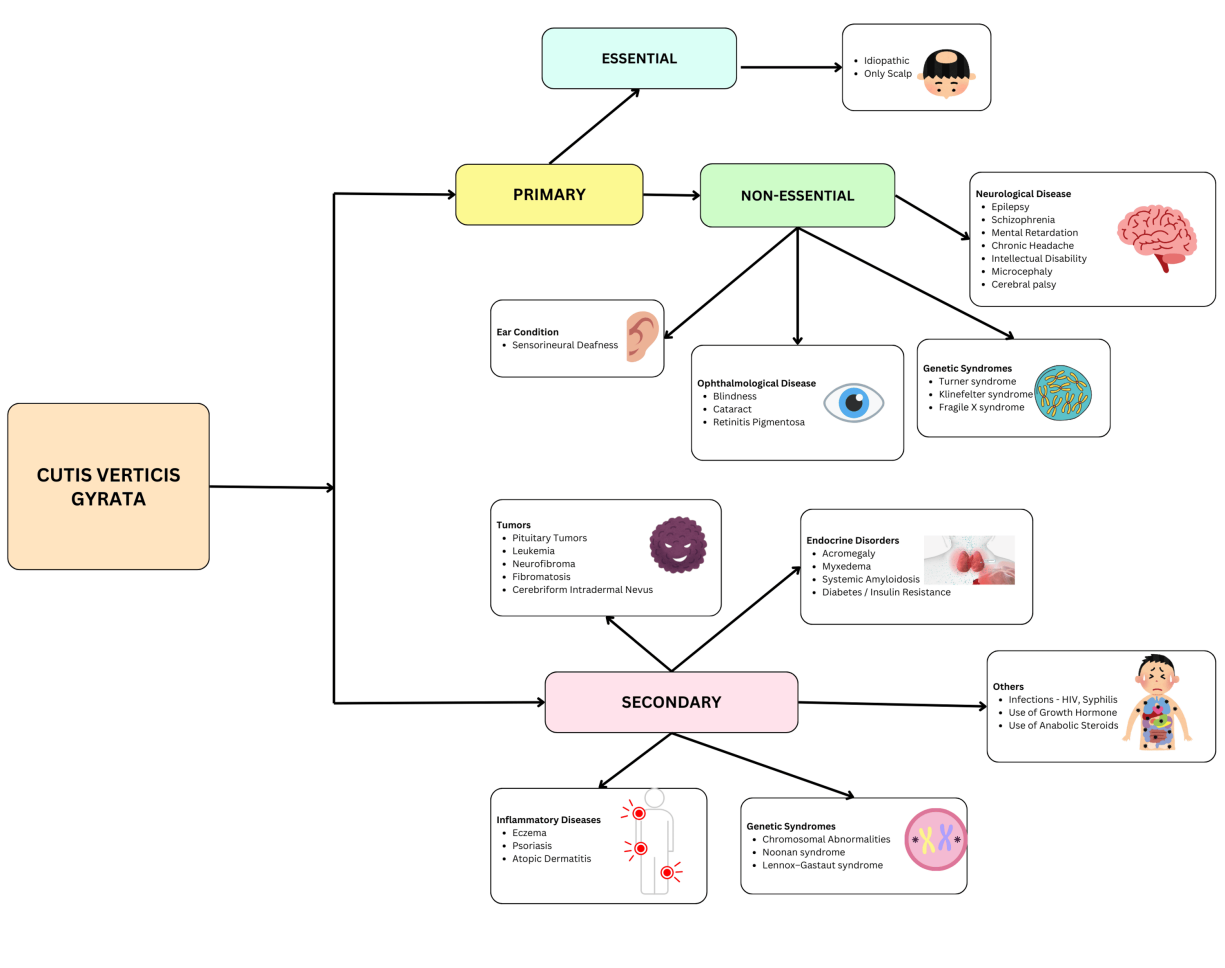
An illustration of various types of cutis verticis gyrata and some known etiological causes Illustration created by Dr. Kevin Morris. Clipart from Canva was used under Canva Free Content License

CVG is a neurocutaneous syndrome in which the scalp tissue folds on itself in the form of sulci and gyri of the brain that run in the anterior-posterior direction. The prognosis is benign; however, it may depend upon the underlying conditions, if any [7]. It is classified into three types: primary essential, primary non-essential, and secondary. Figure 2 illustrates the different types of CVG and their commonly known etiological causes. The primary essential form presents as scalp folds mimicking the cerebral gyri and sulci. It is not known to be associated with neurological or ophthalmological alterations and commonly affects men, usually appearing during or after puberty [8]. Neurological manifestations such as microcephaly, intellectual disability, cerebral palsy, epilepsy, and schizophrenia, as well as ophthalmological manifestations including cataracts and blindness, are usually associated with the primary non-essential type [2]. The secondary form of CVG can occur at any age and affects men and women, with a similar frequency being commonly associated with an underlying cause such as inflammatory dermatoses [9], genetic conditions such as Noonan syndrome [4,9] and Lennox-Gastaut syndrome [9], and endocrine diseases such as acromegaly [10], systemic amyloidosis [11], and intradermal nevus [12].

This case of a female in her mid-30s with CVG and neuropathic pain for the past five to six weeks is rare and novel. The authors assessed the pain using the Leeds Assessment of Neuropathic Symptoms and Signs (LANSS) pain scale [13]. Upon further analysis of the detailed patient history, sensory description of the symptoms, and bedside examination of the sensory dysfunction, it was concluded that the patient had neuropathic pain due to CVG. The addition of neuropathic pain in our case underscores the multifaceted nature of this disorder. The pathophysiology of neuropathic pain in CVG remains poorly understood. However, it is hypothesized that it may potentially involve sensory nerve compression or altered sensory processing due to chronic tissue deformation and hyperproliferation. Management strategies typically focus on pain relief and may include pharmacological drugs such as pregabalin, gabapentin, topical anesthetics, steroids, and non-pharmacological interventions tailored to individual patient needs, such as spinal cord stimulation and physical therapy. In Table 1, we aim to provide a brief overview of the general classification of the types of neuropathic pain based on etiology, clinical characteristics, location, duration, and pathophysiology, with limited examples and currently known treatment strategies [14,15].

**Table 1:** Classification of neuropathic pain and potential treatment strategies.

References	Classification	Types	Examples	Treatment Strategies
Colloca et al., 2017 [14]; Cheng, 2018 [15]	Based on etiology	Central: resulting from the involvement of the brain or spinal cord	Spinal cord injury, multiple sclerosis, Parkinson’s pain, phantom limb pain, post-stroke pain	Antidepressants, antiepileptics (pregabalin, gabapentin), lidocaine, spinal cord stimulation
		Peripheral: resulting from the involvement of peripheral nerve plexus, dorsal root ganglion, or nerve root	Diabetic neuropathy, postherpetic neuralgia, chemotherapy-induced neuropathy	
		Mixed: Resulting from involvement of a combination of neuropathic and nociceptive components	Complex regional pain syndrome type 2 (CRPS-II)	Neural blockade and steroid injections, physical therapy
	Based on clinical characteristics	Spontaneous pain: Occurring without any external trigger	Burning, stabbing, electric shock-like pain	Nerve block, analgesics, antiepileptics, stimulation, physical therapy
		Evoked pain: arises from ordinarily non-painful stimuli	Allodynia, hyperalgesia, hyperpathia	
	Based on location	Focal: affecting facial nerves	Trigeminal neuralgia	Botulinum toxin A
		Multifocal: numerous regions on the body	Multiple nerve damage	Norepinephrine reuptake inhibitor
		Generalized: more widespread pain	Small fiber neuropathy	
	Based on duration	Acute or short-duration pain	Post-surgery, post-injury	Lidocaine
		Chronic or persistent/long-lasting pain	Diabetic neuropathy, post-stroke pain	Antiepileptics (pregabalin, gabapentin), lidocaine, spinal cord stimulation
	Based on pathophysiology	Ectopic activity: requires no stimulation	Abnormal firing activity in primary afferent nerve fibers	Lidocaine
		Increased pain sensitivity	Central/peripheral sensitization causing heightened pain responses	Intrathecal therapies, epidural and transcranial cortical neurostimulation

Neurological complications associated with CVG

Some of the neurological complications associated with CVG identified after a review of contemporary literature are described below.

Mental Retardation

Palo and Iivanainen reported a case of a four-year-old boy with severe mental retardation. There were some other anomalies, including a small asymmetric head, micrognathia, short neck, marmorated skin, thoracic scoliosis, spastic tetraplegia, and epilepsy [16]. A research study by Schepis and Siragusa reported that out of 16 hospitalized developmentally disabled adult patients, two patients were found to be clinically affected with CVG, which was histologically confirmed [17].

Schizophrenia

A study by Schepis and Siragusa reported that out of 49 hospitalized adult schizophrenic patients, one patient was found to be affected with CVG clinically, which was confirmed histologically [17].

Epilepsy

CVG-ID is a term used to describe the association between CVG and intellectual disability (ID). Epilepsy has also been described in CVG-ID. Rattagan et al. reported two cases, including a 33-year-old left-handed male patient and a 36-year-old right-handed male patient, both of whom had drug-resistant epilepsy. These patients also had associated intellectual disability [18]. Striano et al. reported a unique case of CVG-mental deficiency syndrome (CVG-MD) that was associated with bilateral occipital polymicrogyria and drug-resistant epilepsy. A genetic analysis of the chromosomes revealed an increased number of breaks at the 3p14 and 16q23 sites [19].

Headache

Kanwar et al. reported a rare case of a 25-year-old woman who presented with a chronic headache, which was later attributed to increased traction while combing the hair that subsequently led to the development of CVG and non-scarring alopecia [20].

## Conclusions

This case report illustrates a rare presentation of CVG complicated by neuropathic pain, emphasizing the importance of a multidisciplinary approach. The hospital where this case was presented is a tertiary care level urban hospital. Interdisciplinary teamwork, effective communication, and collaboration between various departments such as neurology, dermatology, pathology, and general outpatient clinics are necessary for effective management. In patients with probable neuropathic pain, it is critical to obtain a detailed history, assess the symptoms and signs, analyze the pain using validated tools such as the LANSS pain scale, and then conclude it to be neuropathic pain. Proper treatment strategies including but not limited to timely and correct usage of medications, desensitization techniques such as massages and ice packs, and physical therapy strategies such as yoga and a multidisciplinary approach are warranted in such cases. As far as current literature is concerned, it is the first reported case of neuropathic pain in CVG. The literature review provides elaborate information about the neurological aspects of this benign disease. The need for further research is warranted to elucidate the underlying mechanisms of neuropathic pain in CVG to optimize therapeutic strategies and develop personalized treatment plans for affected individuals.
